# Efficacy of K-wire combined with pull-out wire fixation in the treatment of acute bony mallet finger: a retrospective study

**DOI:** 10.1186/s40001-025-02817-8

**Published:** 2025-07-01

**Authors:** Tianhao Guo, Ruijiao Gao, Aru Zhang, Chenfei Li, Sijia Chen, Jiangbo Bai

**Affiliations:** 1 Department of Hand Surgery, Hebei Medical University Third Hospital, Shijiazhuang, 050051 Hebei People’s Republic of China; 2 Department of Vascular Surgery, Hebei Medical University Third Hospital, Shijiazhuang, 050051 Hebei People’s Republic of China; 3 Hebei Medical University, Shijiazhuang, 050051 Hebei People’s Republic of China

**Keywords:** Bony mallet finger, K-wire fixation, Pull-out wire fixation, DIP joint, Surgical technique, Functional recovery

## Abstract

**Objectives:**

To evaluate the clinical efficacy and safety of a novel surgical technique combining K-wire fixation with pull-out wire fixation for treating acute bony mallet finger.

**Methods:**

This retrospective study included 23 patients with acute bony mallet finger treated between January 2023 and December 2024. Inclusion criteria were injuries within 4 weeks, failed conservative treatment, and fracture fragment size greater than one-third of the articular surface. Surgical details included stabilizing the DIP joint with a K-wire and supplementary fixation with pull-out steel wires to enhance stability. Postoperative outcomes were assessed using the Visual Analog Scale (VAS) score, range of motion (ROM), and Crawford criteria. Data were analyzed using SPSS software.

**Results:**

The mean age of patients was 33.78 ± 10.49 years. All 23 patients experienced no complications. The mean postoperative ROM of the affected DIP joint (75.09 ± 5.32°) was comparable to the healthy side (76.83 ± 5.91°). VAS scores indicated no pain, and Crawford criteria showed excellent or good outcomes in all cases.

**Conclusions:**

The combined K-wire and pull-out wire technique appears to be a safe and effective option for treating acute bony mallet finger, offering stable fixation and good early functional outcomes.

## Introduction 

Bony mallet finger is a common injury characterized by avulsion fractures at the insertion of the extensor tendon on the distal phalanx. This condition results in an inability to extend the distal interphalangeal (DIP) joint actively, often leading to functional impairment and deformity if left untreated [1]. Acute bony mallet finger typically arises from forced flexion of an extended DIP joint, as seen in sports-related trauma or occupational injuries. Early and effective management is essential to restore joint function and prevent long-term complications, such as swan-neck deformity or secondary osteoarthritis [2–5].

Treatment modalities for acute bony mallet finger vary, ranging from conservative approaches, such as splinting, to surgical interventions in cases involving large fracture fragments, joint subluxation, or failure of conservative management [1]. A variety of surgical techniques are available, including isolated K-wire or pull-out steel wire fixation, tension band wiring, and the application of mini bone anchors [1, 6, 7]. Although these approaches have had varying degrees of success, there are non-negligible drawbacks. Yue et al. employed a technique involving two K-wires combined with a rubber band to stabilize the fracture fragment. While this method demonstrated favorable outcomes, it also substantially heightened the risk of skin necrosis due to direct compression of the skin by the K-wire [8]. Tang et al. used two K-wires to compress from the dorsal aspect of the fracture block to treat bony mallet finger. In addition, skin necrosis was observed in 1 out of 17 cases [7]. Damron et al. performed a biomechanical study in which they found that irreversible loss of reduction occurred in all fractures fixed with K-wires, as well as in 60% of tension band wire-fixed fractures and 50% of figure-of-eight wire-fixed fractures [9].

In this study, we propose a novel surgical technique that combines K-wire fixation with pull-out steel wire fixation for the treatment of acute bony mallet finger. This study aims to evaluate the clinical efficacy of this approach, focusing on functional recovery and complication rates. By addressing the limitations of traditional methods, this technique seeks to provide an optimized solution for this complex and challenging condition.

## Methods

### Study design and patients

This retrospective study included patients with bony mallet finger who underwent surgery between January 2023 and January 2024 at our hospital. Patients in this study were required to fulfil all of the following criteria: (1) the time of injury was less than 4 weeks [3, 10, 11]; (2) failure of conservative treatment, defined as lack of improvement in symptoms or joint alignment within 4 weeks of splint immobilization; and (3) fracture mass size greater than one-third of the articular surface. Patients who met any of the following criteria were excluded: (1) the injury was a tendinous mallet finger injury; (2) the fracture was comminuted; (3) the fracture block was not displaced; and (4) patients with serious medical conditions—such as uncontrolled diabetes, active infections, or recent heart attacks—were not included in the study.

### Surgical procedure

The procedure was performed under local or regional anesthesia with the patient in the supine position. The affected hand was placed on a sterile surgical table. After routine disinfection and draping, a C-shaped incision was made over the DIP joint, directly above the fracture site. Expose the fracture fragments, remove the hematoma around the fracture fragments or carefully peel off the surrounding fibrous tissue and scar adhesions, and restore anatomical alignment.

Step 1: A 1.0 mm Kirschner wire (K-wire) is inserted from the fingertip into the middle phalanx to stabilize the distal interphalangeal (DIP) joint in slight hyperextension.

Step 2: Using a 0.8 mm K-wire, two transosseous drill holes are made from dorsal to palmar side in both the distal and middle phalanges (two holes in each phalanx).

Step 3: Four 5 mL syringe needles are inserted through each hole from the palmar to dorsal direction to serve as a guiding channel.

Step 4: Two 0.2 mm steel wires are passed through the lumen of the syringe needles. One end of each wire passes through the distal phalanx and the other through the middle phalanx, crossing dorsally over the fracture fragment to provide compression and stabilization.

Step 5: After the wires are positioned, the syringe needles are withdrawn. The distal end of the K-wire is bent downward, and the external portions of the steel wires are twisted around the K-wire to enhance the strength of the construct.

**Fig. 1:**
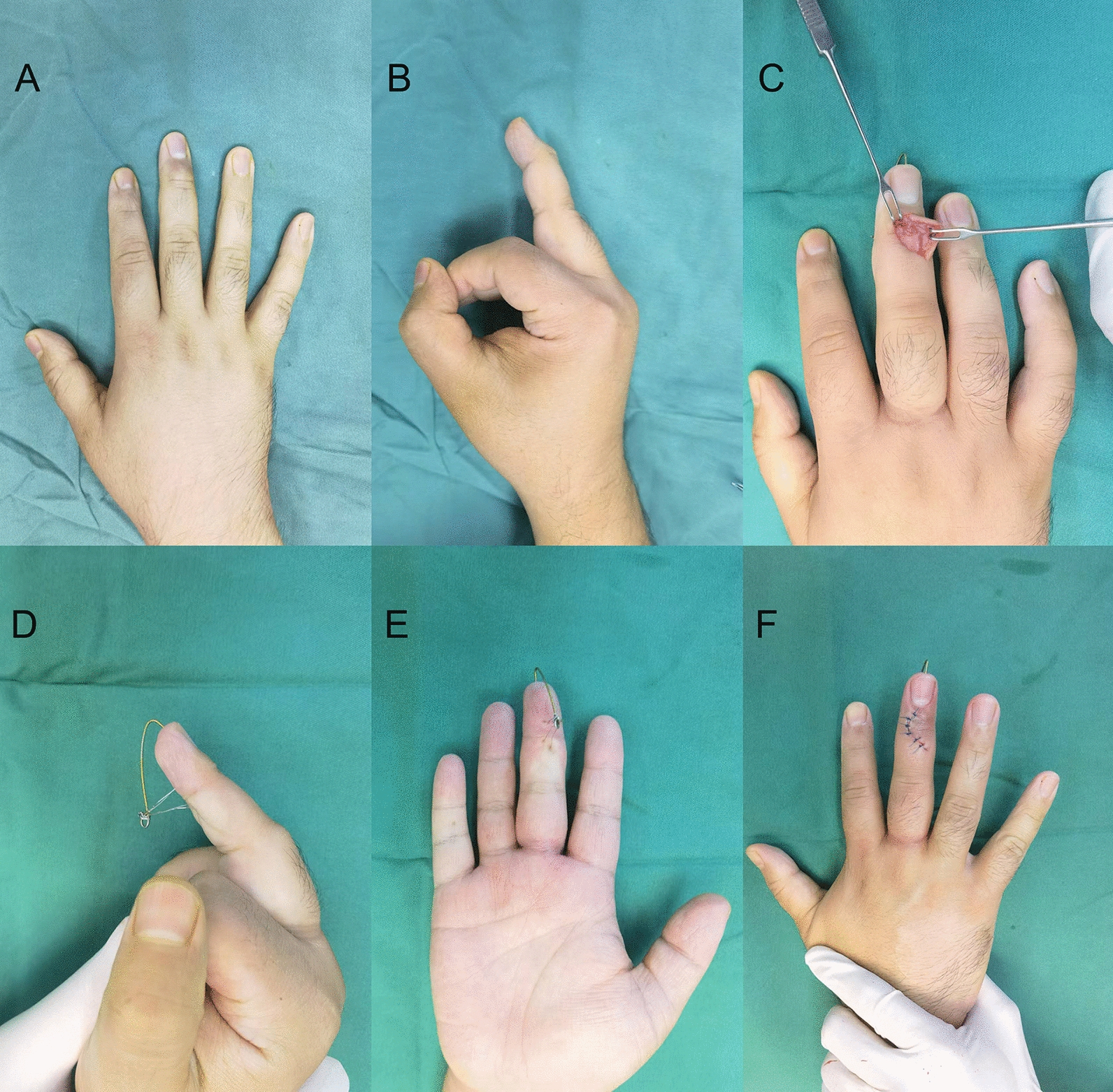
Surgical procedure. **A**, **B** Pre-operative photos. **C** Use of wires to compress the fracture block. **D**–**F** Post-operative photos

**Fig. 2:**
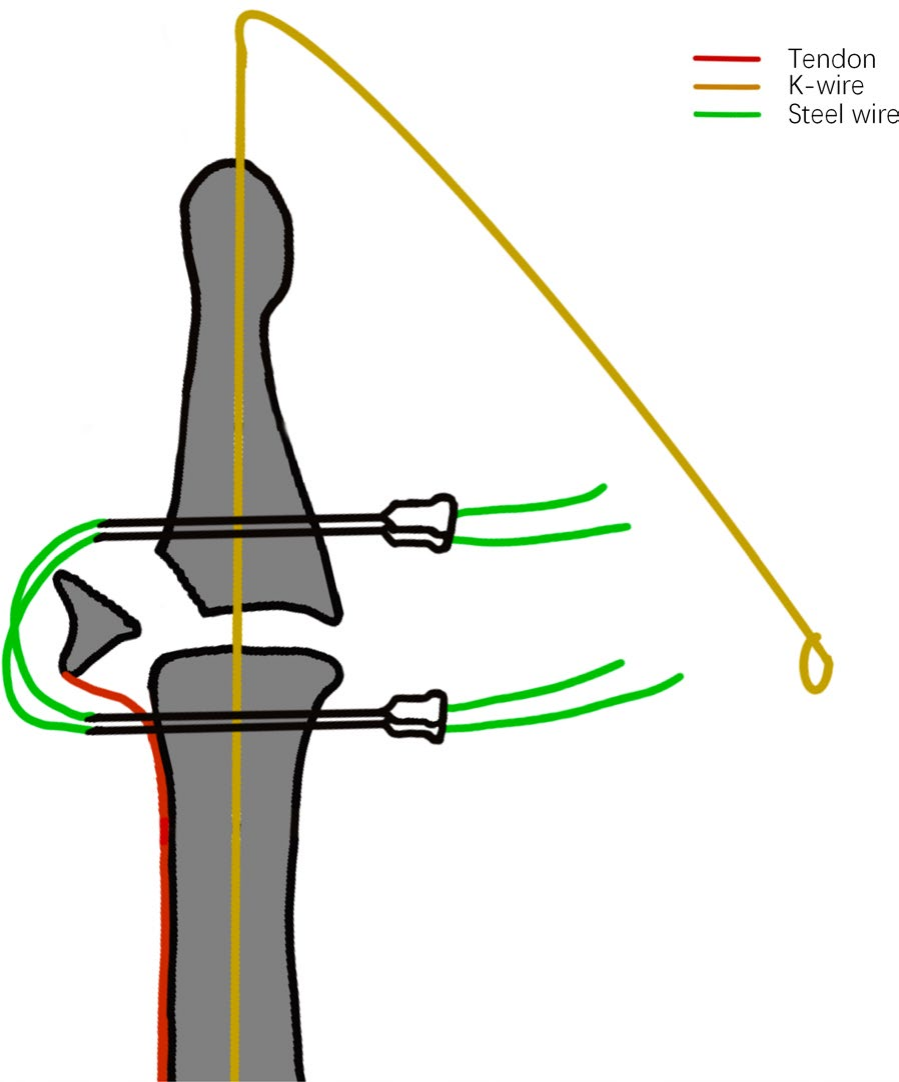
Use of syringe needles to assist in surgery

**Fig. 3:**
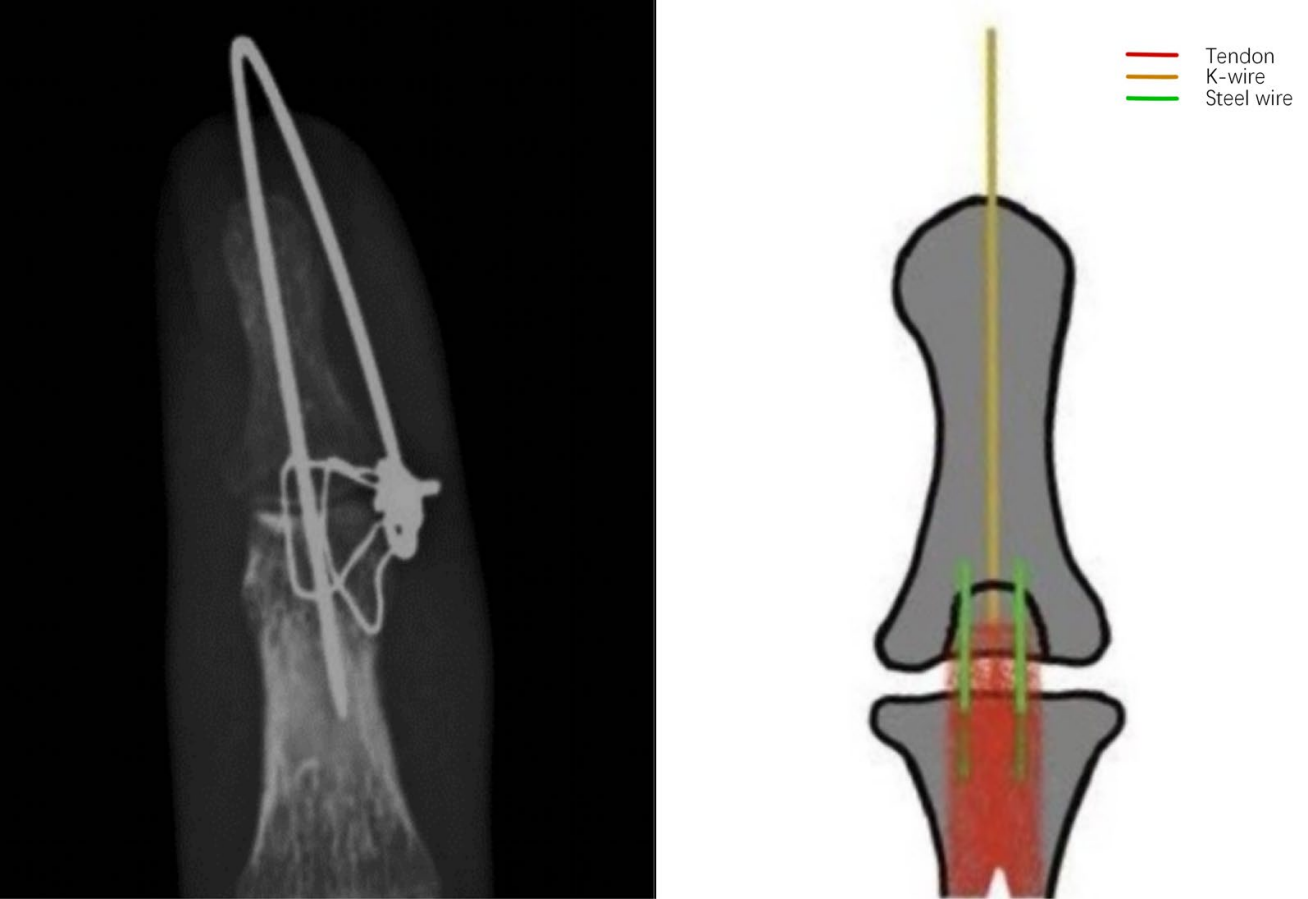
Correct placement of wires and K-wire

**Fig. 4:**
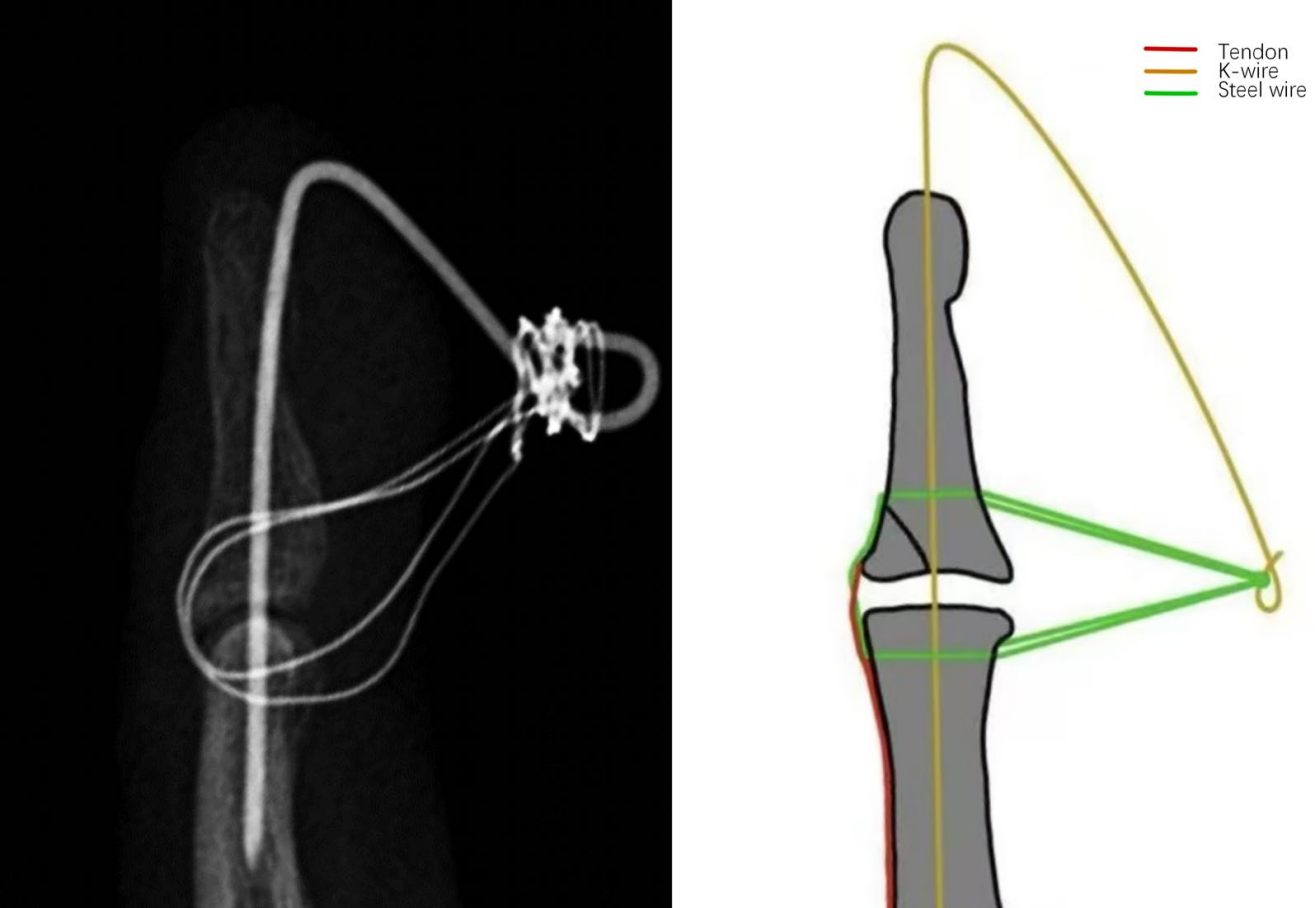
Correct placement of wires and K-wire

Intraoperative X-ray is performed to confirm accurate fracture reduction and the stability of fixation. Finally, the incision is closed using 4–0 non-absorbable sutures, and a sterile dressing is applied. (Figs. 1, 2, 3 and 4).

### Postoperative management

No plaster cast or brace was required to immobilize the affected finger. Antibiotic prophylaxis was administered for 24 h postoperatively. A dressing change was performed on the second postoperative day. The surgical incision and the blood supply to the surrounding skin were carefully observed. Sutures were removed 10–14 days after surgery. K-wire and steel wire were extracted at 6 weeks. Rehabilitation, including gradual range-of-motion exercises, was initiated following wire removal.

### Outcome measures

All patients underwent the first follow-up at 2 weeks postoperatively. The evaluation parameters included wound healing status, fracture healing progress, the presence of skin necrosis, signs of infection at the K-wire insertion site, and any loosening of the steel wire. The second follow-up was conducted at 6 weeks postoperatively, during which the K-wires were removed, and the steel wires were extracted. The primary outcomes assessed included the presence of skin necrosis or infection, and any signs of wound non-healing. Subsequent follow-ups were conducted monthly. The primary parameters collected included finger pain, appearance, and joint function. Finger pain was assessed using the Visual Analog Scale (VAS), with a score greater than 3 indicating the presence of pain. The final follow-up assessments were evaluated based on the Crawford criteria.

### Statistical analysis

Data were analyzed using SPSS version [27.0] (IBM Corp., Armonk, NY, USA). Continuous variables were expressed as Mean ± SD and compared using the independent *t* test or Mann–Whitney *U* test. Categorical variables were presented as frequencies and percentages, analyzed using the chi-square test or Fisher’s exact test. A *p* value < 0.05 was considered statistically significant.

## Results

### Preoperative characteristics

Table 1 summarizes the characteristics of the 23 patients included in this study. The mean age of the patients was 33.78 ± 10.49 years, with a range from 18 to 51 years. There were 15 male and 8 female patients. The affected hand was right in 11 patients and left in 12. According to the Wehbe and Schneider classification method, 4 patients were classified as type 1 A, 2 as type 1B, and 17 as type 2 A or 2B. The mean angle of extension loss was 40.74 ± 6.30 degrees (Table 1).

**Table 1:** Characteristics of the 23 patients

No	Sex	Age (years)	Hand	Classification	Angle of extension loss (degrees)
1	Female	18	Right	1A	32
2	Male	18	Left	1B	40
3	Male	51	Left	2A	45
4	Female	49	Right	2B	48
5	Male	38	Left	1A	35
6	Male	21	Right	2B	38
7	Male	37	Left	2A	42
8	Male	18	Right	2B	35
9	Female	44	Right	1B	31
10	Female	37	Left	2A	40
11	Male	29	Left	2A	39
12	Male	45	Left	2A	45
13	Male	27	Right	1A	35
14	Male	36	Left	2A	42
15	Female	22	Left	2B	46
16	Male	42	Right	2A	40
17	Female	39	Left	2A	43
18	Male	34	Right	2B	50
19	Male	44	Right	2B	48
20	Male	28	Left	2B	55
21	Female	39	Right	1A	30
22	Female	41	Right	2B	38
23	Male	20	Left	2B	40
Mean ± SD		33.78 ± 10.49			40.74 ± 6.30

Classification: Wehbe and Schneider classification method

### Follow-up results

The VAS score, ROM, and Crawford scores in Table 2 are at the last follow-up visit. Table 2 presents the postoperative characteristics of the 23 patients. There were no complications reported in any of the patients. The VAS score for pain was 0 in all patients, indicating no pain postoperatively. Using the ROM of the contralateral (healthy) finger as a reference value, we evaluated the postoperative recovery of the affected finger's ROM. A *t* test revealed a statistically significant difference between the two sides (t = 2.206, P = 0.038). The postoperative ROM of the affected finger (75.09° ± 5.32°) was slightly lower than that of the healthy side (76.83° ± 5.91°), with a small mean difference of 1.74°. The effect size, represented by Cohen’s d = 0.460, indicated a moderate effect. These findings suggest that postoperative functional recovery was relatively good, approaching normal levels, and of clinical significance. According to the Crawford score, which is a commonly used outcome measure for mallet finger injuries, all 23 patients were rated as having excellent or good outcomes. Specifically, 21 patients were rated as excellent, and 2 patient were rated as good (Table 2).

**Table 2:** Postoperative characteristics of 23 patients

No	Complications	VAS	ROM (healthy side)	ROM (affected side postoperatively)	Crawford score
1	No	0	87	85	Excellent
2	No	0	85	70	Good
3	No	0	86	86	Excellent
4	No	0	81	81	Excellent
5	No	0	76	75	Excellent
6	No	0	75	75	Excellent
7	No	0	75	70	Excellent
8	No	0	80	80	Excellent
9	No	0	73	73	Excellent
10	No	0	77	75	Excellent
11	No	0	80	80	Excellent
12	No	0	75	75	Excellent
13	No	0	68	68	Excellent
14	No	0	80	75	Excellent
15	No	0	73	73	Excellent
16	No	0	85	75	Good
17	No	0	71	71	Excellent
18	No	0	79	79	Excellent
19	No	0	76	76	Excellent
20	No	0	70	70	Excellent
21	No	0	80	80	Excellent
22	No	0	65	65	Excellent
23	No	0	70	70	Excellent
Mean ± SD			76.83 ± 5.91	75.09 ± 5.32^a^	

VAS: Visual Analogue Scale; ROM: Range of Motion

^a^The ROM of the healthy and affected side showed statistically significant difference (*P* = 0.038)

**Fig. 5:**
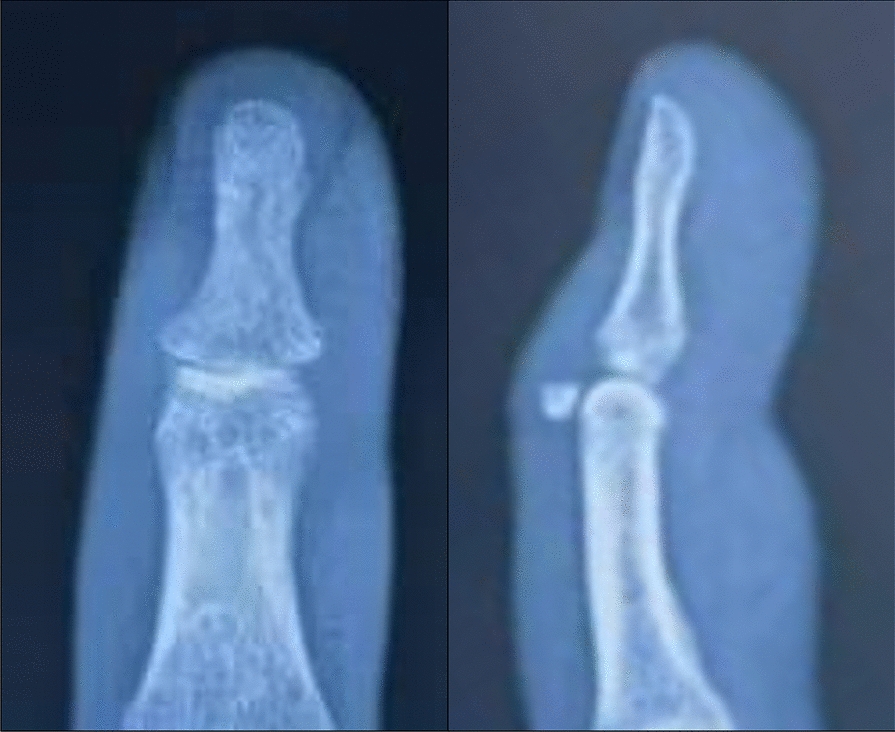
Pre-operative X-ray photos of the hand

**Fig. 6:**
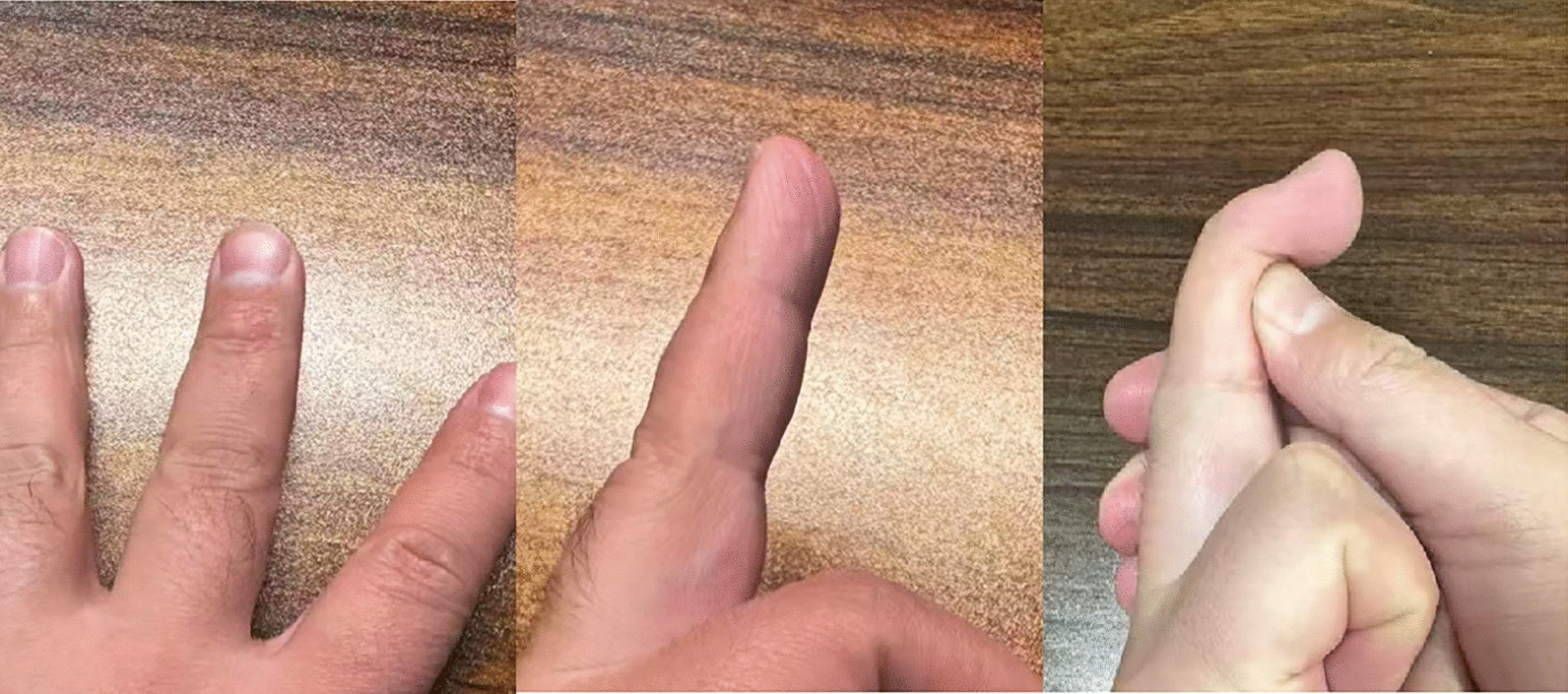
Follow-up of one patient 6 months after surgery

Figure 5 presents a preoperative radiograph of the patient, while Fig. 1 depicts an intraoperative photograph taken at the time of surgery. Figure 6 illustrates the patient's condition at 6 months postoperatively (Figs. 1, 5 and 6).

No complications, such as skin necrosis, infection, impaired wound healing, or traumatic osteoarthritis, were observed in any of the patients.

## Discussion

This study provides a comprehensive evaluation of the K-wire combined with pull-out wire fixation for treating acute bony mallet finger. The findings highlight the efficacy of this technique in addressing the limitations of existing surgical methods by achieving stable fixation, excellent functional recovery, and minimal complications.

Currently, there are a variety of treatments for bony mallet fingers, but the best treatment remains controversial [12]. The main surgical methods include K-wire fixation, percutaneous screw fixation, microplate fixation, pulling technique, and K-wire compression technique. Although these approaches have had varying degrees of success, there are non-negligible drawbacks [8, 13, 14]. Zhang et al. previously employed a technique in which double K-wires were passed directly through the fracture site to achieve reduction. However, this method may lead to fragmentation of the fracture fragment during the insertion of the K-wires and is less suitable for smaller fracture fragments [14]. A new extension-block pinning technique was described by Çapkın et al. to treat bony mallet finger [15]. However, it is not feasible when the bone fragment rotates. Hofmeister et al. managed displaced bony mallet fingers using an extension block pin combined with transarticular fixation of the DIP joint. Among 24 cases, the mean extension loss was reported to be 4° at a follow-up of 1 year or longer. Nevertheless, this approach has been associated with an extension lag of up to 20° in some cases [16]. In the 11 cases we reported, we did not find any complications, such as skin necrosis, K-wire's needle tract infection, infection around the pull-out wire, failed reset, or prominent dorsal bump. Our findings suggest that this technique may offer greater reliability and fewer complications compared to traditional methods, as evidenced by the absence of complications in our series of 11 cases.

In our surgical procedure, there are several key points that should be noted. First, the wires should be orientated parallel to the longitudinal axis of the finger. The wire should be tunneled through the middle phalanx as close to the distal end as possible and positioned to pass through the extensor tendon near the fracture fragment, thereby minimizing potential damage to the extensor tendon (Fig. 3). Second, the wire should pass through the finger as vertically as possible to minimise damage to the soft tissues of the finger from the shear forces generated by the angle of the wire (Fig. 4). Third, using a 5 mm syringe needle to assist in passing the wire through the phalanx is a feasible method, and the spacing between the wire entry points should not be too wide to avoid damaging the arteries and nerves. Fourth, the distal finger can be maximally flexed and traction applied distally, allowing exposure of the residual articular surface at the base of the phalanx. The insertion point is located on the articular surface, and the wire is advanced bidirectionally through the double-headed K-wire. This technique ensures that the K-wire avoids the fracture site, thereby preventing interference with fracture reduction.

The dorsal steel wires are completely buried beneath the skin. Once the fracture has met the clinical criteria for healing, the wires can be removed without the need for an additional incision. The steel wire is first cut at the palmar side, close to the skin surface. The operative area is then thoroughly disinfected, and the wire is gently withdrawn from another side. The method allows for safe, minimally invasive wire removal while avoiding further trauma to the skin and extensor tendon.

Injuries within 4 weeks of onset were considered acute. However, we recognize that tissue response within this period is progressive. Typically, during the first week after injury, a small hematoma may be present at the fracture site, and the surrounding soft tissues remain relatively clear, which facilitates surgical exposure and reduction. From approximately 1 to 4 week post-injury, fibrous scar tissue and adhesions begin to develop gradually, which may obscure anatomical landmarks and increase the difficulty of accurate reduction. These pathological changes become more prominent over time and are important considerations in planning surgical management.

The biomechanical advantage of combining K-wire with pull-out wires fixation lies in its ability to provide multidirectional stabilization. The pull-out wire provides additional compression across the fracture site, improving coaptation and resistance to shear forces. Our approach combines K-wire and pull-out wire fixation, leveraging the strengths of both techniques. This hybrid method ensures robust fixation and compression, minimizing fragment displacement and enhancing stability. This technique demonstrates superior functional outcomes and a low complication rate.

This study has certain limitations. Although data collection was conducted prospectively, the analysis itself was retrospective. Future investigations should emphasize prospective randomized controlled trials to enhance the reliability and validity of the results. Second, this study did not include a comparative analysis with other surgical techniques for treating bony mallet finger. Further comparisons with alternative procedures are necessary before this approach can be widely promoted. Moreover, the single-center nature of this study may restrict the broader applicability of the results. To confirm these findings across different populations and clinical environments, future multicenter research is warranted. In addition, although this study offers meaningful insights into short-term outcomes, extended follow-up is essential to evaluate the persistence of symptom relief and the long-term recovery of function.

## Conclusion

The combined K-wire and pull-out wire fixation technique may serve as a viable option for treating acute bony mallet finger, demonstrating stable fixation and encouraging short-term functional outcomes with a low complication rate. However, further studies with larger sample sizes and longer follow-up are needed to confirm its efficacy and generalizability.

## Data Availability

The datasets used and/or analysed during the current study are available from the corresponding author on reasonable request.

## References

[1] Bendre AA, Hartigan BJ, Kalainov DM. Mallet finger. J Am Acad Orthop Surg. 2005;13:336–44. 10.5435/00124635-200509000-00007. 16148359 10.5435/00124635-200509000-00007

[2] Giddins GE. Bony mallet finger injuries: assessment of stability with extension stress testing. J Hand Surg Eur. 2016;41:696–700. 10.1177/1753193416647307. 10.1177/1753193416647307 27215226

[3] Alla SR, Deal ND, Dempsey IJ. Current concepts: mallet finger. Hand (N Y). 2014;9:138–44. 10.1007/s11552-014-9609-y. 24839413 10.1007/s11552-014-9609-y PMC4022957

[4] Moradi A, Kachooei AR, Mudgal CS. Mallet fracture. J Hand Surg Am. 2014;39:2067–9. 10.1016/j.jhsa.2014.06.022. 25135247 10.1016/j.jhsa.2014.06.022

[5] Lin JS, Samora JB. Outcomes of splinting in pediatric mallet finger. J Hand Surg Am. 2018;43(1041):e1041-1041.e1049. 10.1016/j.jhsa.2018.03.037. 10.1016/j.jhsa.2018.03.037 29776724

[6] Jupiter JB, Sheppard JE. Tension wire fixation of avulsion fractures in the hand. Clin Orthop Relat Res. 1987;214:113–20. 3791732

[7] Tang J, Wu K, Wang J, Zhang J. Open reduction and compression with double Kirschner wires for the treatment of old bony mallet finger. J Orthop Surg Res. 2019;14:459. 10.1186/s13018-019-1513-2. 31864378 10.1186/s13018-019-1513-2 PMC6925853

[8] Yue Z, Mo Y, Xiong Z, Tang Y. New technique for the treatment of fresh bony mallet finger: a retrospective case series study. Front Surg. 2023;10:1127827. 10.3389/fsurg.2023.1127827. 37065995 10.3389/fsurg.2023.1127827 PMC10090349

[9] Damron TA, et al. Biomechanical analysis of mallet finger fracture fixation techniques. J Hand Surg Am. 1993;18:600–7. 10.1016/0363-5023(93)90298-h. 8349965 10.1016/0363-5023(93)90298-H

[10] Yoon JO, Baek H, Kim JK. The outcomes of extension block pinning and nonsurgical management for mallet fracture. J Hand Surg Am. 2017;42(387):e381-387.e387. 10.1016/j.jhsa.2017.02.003. 10.1016/j.jhsa.2017.02.003 28274605

[11] Asano K, Inoue G, Shin M. Treatment of chronic mallet fractures using extension-block Kirschner wire. Hand Surg. 2014;19:399–403. 10.1142/s0218810414500348. 25288289 10.1142/S0218810414500348

[12] Lamaris GA, Matthew MK. The diagnosis and management of mallet finger injuries. Hand (N Y). 2017;12:223–8. 10.1177/1558944716642763. 28453357 10.1177/1558944716642763 PMC5480656

[13] Ishiguro T, Imai N, Tomatsu T, Noguchi T, Hashizume N. A new method of closed reduction using the spring action of Kirschner wires for fractures of the tibial plateau–a preliminary report. Nihon Seikeigeka Gakkai Zasshi. 1986;60:227–36. 3722970

[14] Zhang H, Du S, Zhu J. A novel surgical technique for direct fixation of bony mallet finger with K-wires. J Hand Surg Eur. 2024. 10.1177/17531934241299214. 10.1177/17531934241299214 39579372

[15] Çapkın S, Buyuk AF, Sürücü S, Bakan OM, Atlihan D. Extension-block pinning to treat bony mallet finger: is a transfixation pin necessary? Ulus Travma Acil Cerrahi Derg. 2019;25:281–6. 10.5505/tjtes.2018.59951. 31135948 10.5505/tjtes.2018.59951

[16] Hofmeister EP, Mazurek MT, Shin AY, Bishop AT. Extension block pinning for large mallet fractures. J Hand Surg Am. 2003;28:453–9. 10.1053/jhsu.2003.50089. 12772104 10.1053/jhsu.2003.50089

